# Brain Resting-State Network Alterations Associated With Crohn's Disease

**DOI:** 10.3389/fneur.2020.00048

**Published:** 2020-02-18

**Authors:** Jennifer Kornelsen, Alyssia Wilson, Jennifer S. Labus, Kelcie Witges, Emeran A. Mayer, Charles N. Bernstein

**Affiliations:** ^1^Department of Radiology, University of Manitoba, Winnipeg, MB, Canada; ^2^IBD Clinical and Research Centre, University of Manitoba, Winnipeg, MB, Canada; ^3^Department of Internal Medicine, University of Manitoba, Winnipeg, MB, Canada; ^4^G. Oppenheimer Family Center for Neurobiology of Stress and Resilience, David Geffen School of Medicine at UCLA, Los Angeles, CA, United States

**Keywords:** Crohn's disease (CD), resting-state networks (RSNs), functional connectivity (FC), functional magnetic resonance imaging (fMRI), inflammatory bowel disease (IBD)

## Abstract

Inflammatory bowel disease (IBD) is a chronic disease that is associated with aspects of brain anatomy and activity. In this preliminary MRI study, we investigated differences in brain structure and in functional connectivity (FC) of brain regions in 35 participants with Crohn's disease (CD) and 21 healthy controls (HC). Voxel-based morphometry (VBM) analysis was performed to contrast CD and HC structural images. Region of interest (ROI) analyses were run to assess FC for resting-state network nodes. Independent component analysis (ICA) identified whole brain differences in FC associated with resting-state networks. Though no structural differences were found, ROI analyses showed increased FC between the frontoparietal (FP) network and salience network (SN), and decreased FC between nodes of the default mode network (DMN). ICA results revealed changes involving cerebellar (CER), visual (VIS), and SN components. Differences in FC associated with sex were observed for both ROI analysis and ICA. Taken together, these changes are consistent with an influence of CD on the brain and serve to direct future research hypotheses.

## Introduction

Inflammatory bowel disease (IBD) consists of two main conditions, Crohn's disease (CD) and ulcerative colitis (UC) (1), which are rising in prevalence around the world (2). The main working etiologic hypothesis is that in persons genetically predisposed to develop CD, the gut microbiome triggers a maladaptive immune response causing chronic gut inflammation (1, 3). Patient symptoms may vary in severity and activity, but normally include abdominal pain, diarrhea, and weight loss, which can greatly affect quality of life and mental health (4). Chronic disorders, such as CD not only affect physical health, but also mental health, thereby increasing the chances of acquiring comorbid mood disorders including depression or anxiety (5–7). In fact, it has also been shown that persons with CD are more likely to have depression or anxiety antedating CD diagnosis by years, suggesting possible shared risk factors for both chronic mental health disorders and CD (8, 9). Physiologically, as a threat to homeostasis, there are a number of mechanisms by which stress may impact the gastrointestinal tract (10). Mood and CD demonstrate a reciprocal effect on stress and flare-ups through the brain–gut axis (11). Stress (12–14), pain (15–18), and mood disorders (15, 19–21) are all factors that influence structure and function of the brain.

Investigations into altered brain structure and function in CD have increased; however, the existing body of literature is limited and there is a lack of consistency among the techniques used and the results reported that prohibits any key brain alterations to be identified. Studies of brain structure in CD may be in agreement on one result, altered gray matter (GM) of the superior frontal gyrus, although laterality and direction of alteration are inconsistent (22–24). Studies of brain function in CD have reported numerous FC alterations with very little overlap (25–32) aside from some agreement on changes in brain networks or regions involved in executive function and/or default mode (26, 30, 32). Our study aimed to identify brain structural and functional changes in CD as compared to HC. In an attempt to isolate the relationship of CD and the brain in the absence of psychiatric comorbidity, the study exclusion criteria include depression and anxiety. Differences between groups were expected for both GM volume and functional connectivity (FC). Sex differences in FC were also expected. Voxel-based morphometry was used to assess structural difference in GM volume, and both region of interest (ROI) analysis and independent component analysis (ICA) were used to detect differences in the FC of resting**-**state networks (RSNs). By employing three methods of assessing change in brain structure or function in the same study participants, this preliminary approach will identify changes in the brain that are associated with CD and provide hypothesis-formation guidance for future work.

## Methods

### Participants

This study was approved by the University of Manitoba Research Ethics Board and UCLA Institutional Review Board and all participants signed an informed consent form. For this study, 35 individuals with CD [18 males and 17 females, mean age 32.6 ± 11.4 years, body mass index (BMI) 25.5 ± 4.4; 17 at UCLA and 18 at UM] were recruited. Patients had been previously diagnosed with CD and were recruited through their previous involvement in a local longitudinal IBD cohort study, a provincial-based research registry, regional gastroenterology clinics, and via website (ibdmanitoba.org). We reviewed medical records to confirm IBD diagnoses and contacted physicians for additional information as needed. Twenty-one healthy controls (HC) (14 males, 7 females, mean age 26.9 ± 10.0 years, BMI = 25.5 ± 4.9; 16 at UCLA and 5 at UM) with no history of gastrointestinal diagnosis or symptoms were enlisted.

The inclusion criteria for CD patients included right-handed, ambulatory English-speaking adults aged between 18 and 55 years. Exclusion criteria included non-CD-related structural abnormalities of the gastrointestinal tract, additional gastrointestinal disease or non-CD-related surgery, or involvement in an ongoing clinical trial. Inclusion criteria for HC were age between 18 and 55 years, and no gastrointestinal history or a diagnosis or symptoms of irritable bowel syndrome (IBS) meeting the ROME III diagnostic criteria. Exclusion criteria for all subjects included left-handedness, illicit drug use, any neurological or psychiatric conditions (including depression and anxiety), morbid obesity (BMI > 35), post-menopausal status, or MRI incompatibility.

### Study Design

Before imaging, all patients and controls provided written informed consent. Age, sex, and BMI were recorded for all participants. Patients completed the Harvey–Bradshaw questionnaire to determine symptomatic disease activity (33). Patients were considered to have active disease with a score of 5 or higher. Bowel habit was classified as abnormal for constipation, diarrhea, alternating, mixed, or unspecified, and normal otherwise. Patients were phenotyped using the Montreal classification system (34) to determine location [L1 (ileum), L2 (colonic), L3 (ileocolonic), and L4 (only upper disease)] and disease behavior [B1 (only inflammatory disease), B2 (fibrostenosing disease), B3 (fistula), P (perianal disease)]. Patient use of biologic therapy (use of adalimumab or infliximab) and of non-biologic therapy was documented. Disease duration in years was recorded. All study participants were screened for MRI safety before entering the scanner.

### MRI Acquisition

Using a Siemens Magnetom Verio 3T at UM and a Siemens Magnetom Trio 3T at UCLA and 12-channel head coil [Erlangen, Germany], we acquired T1-weighted three-dimensional magnetization-prepared rapid gradient echo (MPRAGE) scans. The imaging protocol file was identical at each site and quality assurance checks were conducted during separate pilot testing prior to study commencement. Data were acquired with the following parameters: TR = 2,300 ms, TE = 3.02 ms, field of view = 256 × 240 mm, matrix = 256 × 256, voxel size was 1.0 × 1.0 × 1.0 mm^3^, flip angle 9°, and slices = 240. Functional data were acquired using T2^*^-weighted whole brain echo planar imaging (EPI) sequence with TR = 2,000 ms, TE = 28 ms, field of view = 220 × 220 mm, matrix = 64 × 64, flip angle = 77°, and slices = 37 with 4 mm slice thickness and 12% distance factor, with participants' eyes closed.

### Voxel-Based Morphometry Analysis

To assess GM volume, structural MRI data were preprocessed and analyzed using SPM12 software (http://www.fil.ion.ucl.ac.uk/spm) and CAT12 toolbox, which was run on MATLAB (version 2018a; Mathworks Inc., Natick, MA USA). T1-weighted images were segmented into GM and white matter (WM). Default settings for the CAT12 segmentation pipeline were used, including affine regularization with the ICBM space template-European brains and spatial registration used Diffeomorphic Anatomical Registration Through Exponentiated Lie Algebra (DARTEL) (35) IXI555_MNI152 template (http://www.brain-development.org) with an isotropic voxel size of 1.5 mm. Modulated normalized GM and WM images were saved, with scaling by the Jacobian determinants. GM segments were checked for homogeneity and images were smoothed in SPM12 with an 8-mm full width at the half maximum (FWHM) Gaussian kernel. Total intracranial volume (TIV) was estimated and saved.

Using SPM12 to examine GM volume differences between CD patients and HCs, a two-sample *t*-test with an absolute masking threshold of 0.2 controlled for TIV, age, sex, BMI, and acquisition site was run. The statistical threshold was set at *p* < 0.05, FDR-corrected at the cluster level, with a minimum cluster size of 5 voxels. In the case that significant differences between groups were detected, additional analyses were planned for further investigation of significant structural differences within the CD group by covarying disease duration with volumetric measures, and performing two-sample *t*-tests of Montreal classification L1 vs. L2 and L3 combined, and Montreal classification B1 vs. B2 and B3 combined.

### Functional Pre-processing

Resting**-**state FC was assessed using the CONN toolbox Version 17.f (36) to preprocess and analyze the functional imaging data. The functional data were functionally realigned and unwarped; translated by centering to (0,0,0) coordinates; slice time corrected; scrubbed with ART-based identification for outlier scans; segmented into GM, WM, and CSF and normalized to the Montreal Neurological Institute (MNI) template; and smoothed using an 8-mm Gaussian kernel. Structural data were translated by centering to (0,0,0) coordinates, segmented into GM, WM, and CSF, and normalized to the MNI template. Artifact detection was run using the ART toolbox. During the de-noising processes, WM, CSF, and outliers detected by the ART toolbox were entered into the linear regression as confounding effects. Subject motion correction was performed via realignment parameters entered in the linear regression of confounding effects (first-order derivatives, no polynomial expansion). No significant differences between groups were found for realignment average raw scores (1-tailed *p* = 0.176) or average framewise (scan-to-scan) differences (1-tailed *p* = 0.170). Linear de-trending was performed, and the default band-pass filter of [0.008 0.09] Hz was applied.

### ROI Analysis

Functional analysis was performed using the ROI-to-ROI function in the CONN toolbox using the General Linear Model (GLM, correlation analysis settings, and no weighting applied). The ROI-to-ROI analysis was run using CONN RSN nodes (36), which include 32 seeds/targets [Default Mode Network (DMN): medial pre-frontal cortex (MPFC), precuneus cortex (PCC), bilateral lateral parietal (LP); Sensorimotor Network (SMN): Superior, bilateral Lateral; Visual Network (VIS): Medial, Occipital, bilateral Lateral; Salience Network (SN): anterior cingulate cortex (ACC), bilateral anterior insula (AI), rostral pre-frontal cortex (RPFC), and supramarginal gyrus (SMG); Dorsal Attention Network (DA): bilateral frontal eye field (FEF) and intraparietal sulcus (IPS); Fronto-parietal Network (FP): bilateral lateral pre-frontal cortex (LPFC) and posterior parietal cortex (PPC); Language Network (LAN): bilateral inferior frontal gyrus (IFG) and posterior superior temporal gyrus (pSTG); and Cerebellar Network (CER): Anterior, Posterior].

### Independent Component Analysis

Using default settings for CONN 17.f, the ICA was run with a FastICA for estimation of independent spatial components and GICA1 back-projection for individual subject level spatial map estimation. Dimensionality reduction was set to 64 and number of components was set to 31. The number of components was estimated using GIFT 4.0b software, using minimum description length (MDL) criteria, to estimate components for each individual separately, and then compute the estimated number of components for the entire data set using the mean, median, and maximum standard deviation of individual results. The correlational spatial match-to-template approach was used in CONN to identify within each component the brain RSNs for the cerebellar (CER), default mode (DMN), sensorimotor (SMN), frontoparietal (FP), dorsal attention (DA), salience (SN), language (LAN), and visual (VIS) networks.

### Statistics

Between-subjects [CD (1) HC (−1)] contrasts were run for both ROI and ICA analyses, controlling for age, sex, BMI, and acquisition site. For the ROI analysis, all network nodes were used as both sources and targets, and ROI-to-ROI connections were set to a threshold by intensity of two-sided FDR-corrected *p* < 0.05. ICA results were displayed at *p* < 0.001 uncorrected and a cluster-wise threshold of *p* < 0.05, FDR-corrected. Additional analyses were run to explore differences in FC in CD patients. The relationship between FC and disease duration in years was assessed with linear regression, and Montreal classification L1 vs. L2 and L3 combined as well as Montreal classification B1 vs. B2 and B3 combined, while controlling for age, sex, BMI, and acquisition site were assessed with two-tailed two-sample *t*-tests. The *t*-test of L1 vs. L2/L3 combined compares isolated small bowel disease to small bowel plus colon or colon alone, to see if colon involvement is associated with different outcomes, whereas B1 vs. B2/B3 combined compares pure inflammatory vs. complicated (stricture and/or fistula) disease. Sex differences were investigated by contrasting [CD females (1) CD males (−1)], [CD females (1) HC females (−1)], and [CD males (1) HC males (−1)], while controlling for age, BMI, and acquisition site.

### *Post-hoc* Multimodal Analysis

Following the structural and functional data analyses, a multimodal analysis was performed using MRIcroGL version 1.2.20190902. The areas of significantly different FC identified by the ICA were summed such that the five components formed a single layer representing the ICA results. Similarly, the significant differences between groups identified in the ROI-to-ROI analysis were summed into a single layer. The ICA, ROI-to-ROI, and spm152 atlas layers were overlaid. The Additive Overlay Blending option was selected to identify areas of spatial overlap. Although the VBM analysis did not produce significant results when corrected for multiple comparisons, the spmT map of the contrast between groups before correction for multiple comparisons was used as a layer to compare with the ICA and ROI-to-ROI layers independently. Finally, all three layers were overlaid with the spm152 template and assessed for areas of spatial overlap.

## Results

### Demographic Results

As seen in Table 1, no significant differences were found between CD patients and HC for sex, age, or BMI. However, bowel habits between the two groups were significantly different.

**Table 1:** Demographics and relevant clinical data for all subjects.

	**CD (*n* = 35)**	**HC (*n* = 21)**	***p*-value**
Sex (male/female)	18/17	14/7	0.273
Age (years)	32.6 ± 11.4	26.9 ± 10.0	0.066
BMI	27.1 ± 4.4	25.5 ± 4.9	0.801
Harvey–Bradshaw (active/inactive)	7/28	–	–
Bowel habits (normal/abnormal)	14/21	21/0	<0.001
Disease duration (years)	11.3 ± 7.1	–	–
Montreal classification			
Location			
L1	18		
L2	3	–	–
L3	13	–	–
L4*	3	–	–
Behavior			
B1	20	–	–
B2	7	–	–
B3	10	–	–
P	5	–	–
IBD medications (biologics/no biologics)	13/22	–	–

Values for age, BMI, and disease duration are presented as mean values and standard deviations. Values for sex, bowel habits, Harvey–Bradshaw, Montreal classification, and IBD medications are presented as number of subjects in that category. BMI, body mass index; L1, ileum; L2, colonic; L3, ileocolonic; L4^*^, upper gastrointestinal tract disease (participants could have L4 disease and any of L1, L2, and L3 as well); B1, only inflammatory; B2, stricture in bowel; B3, fistula; P, perianal disease; IBD, inflammatory bowel disease.

### Voxel-Based Morphometry Results

No GM volume differences were found between CD patients and HCs when using FDR correction for multiple comparisons. As no differences were found between the groups, no further analyses were run.

### ROI Results

**Figure 1:**
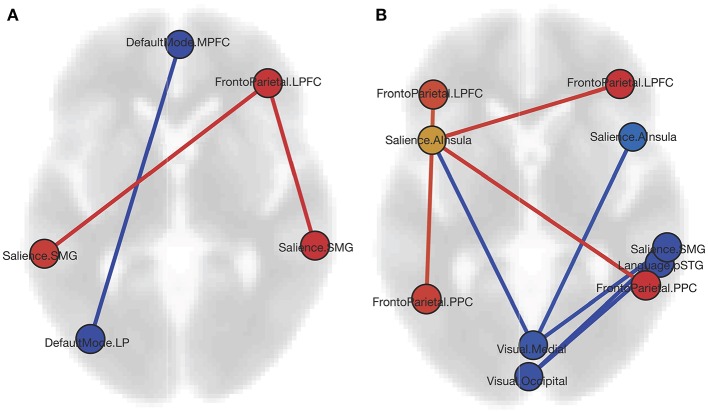
**(A)** Graphic of ROI-to-ROI contrast showing nodes with increased FC (red) and decreased FC (blue) for the CD group as compared to HCs. **(B)** Graphic of ROI-to-ROI contrast showing nodes with increased FC (red), decreased FC (blue), and both increased and decreased FC (yellow) for the CD females as compared to the HC females. Both graphics are displayed in axial orientation with the anterior aspect toward the top and right on the right, with ROI-to-ROI connection threshold set by intensity at FDR *p* < 0.05, two-sided (MPFC, medial pre-frontal cortex; LPFC, lateral pre-frontal cortex; SMG, supramarginal gyrus; LP, lateral parietal; AI, anterior insula; PPC, posterior parietal cortex; STG, superior temporal gyrus).

The ROI analysis shows that CD patients have reciprocally increased FC between the right lateral pre-frontal cortex (LPFC) of the FP network and bilateral supramarginal gyrus nodes of the SN, and decreased FC between the medial pre-frontal cortex (MPFC) and the left lateral parietal (LP) node of the DMN (Table 2, Figure 1A). Bivariate correlations between right LPFC and bilateral SMG indicate that HC had negative FC whereas CD had positive FC between nodes. FC was less positive for CD than HC for the MPFC and left LP nodes.

**Table 2:** ROI-to-ROI results showing functional connectivity differences between Crohn's disease patients and healthy controls (CD > HC) controlling for age, sex, BMI, and acquisition site followed by results of contrast between patient females and healthy females (CDF > HCF) controlling for age, BMI, and acquisition site (threshold ROI-to-ROI connections by intensity, FDR *p* < 0.05, two-sided).

**Seed**	**Target**	***t***	***p***	***r* CD**	***r* HC**
**CD > HC**					
Lateral pre-frontal cortex R	Supramarginal gyrus L	3.53	0.019	0.07	−0.10
	Supramarginal gyrus R	3.43	0.019	0.10	−0.11
Supramarginal gyrus L	Lateral pre-frontal cortex R	3.53	0.028	0.07	−0.10
Supramarginal gyrus R	Lateral pre-frontal cortex R	3.43	0.038	0.10	−0.11
Medial pre-frontal cortex	Lateral parietal L	−3.93	0.008	0.19	0.39
Lateral parietal L	Medial pre-frontal cortex	−3.93	0.008	0.19	0.39
**CDF > HCF**					
Anterior insula L	Lateral pre-frontal cortex L	3.01	0.004	0.07	−0.03
	Lateral pre-frontal cortex R	3.03	0.004	0.00	−0.13
	Posterior parietal cortex L	3.23	0.017	0.00	−0.07
	Posterior parietal cortex R	3.52	0.001	0.06	−0.05
	Visual medial	−3.81	0.001	−0.14	−0.05
Visual occipital	Supramarginal gyrus R	−4.69	0.001	−0.11	−0.07
	Superior temporal R	−3.51	0.015	−0.06	0.05
Supramarginal gyrus R	Visual occipital	−4.69	0.001	−0.11	−0.07
	Visual medial	−3.20	0.036	−0.12	−0.04
Visual medial	Anterior insula L	−3.81	0.012	−0.14	−0.05
	Anterior insula R	−3.57	0.012	−0.10	−0.05
	Supramarginal gyrus R	−3.20	0.024	−0.12	−0.04
Anterior insula R	Visual medial	−3.57	0.025	−0.10	−0.05
Superior temporal R	Visual occipital	−3.51	0.029	−0.06	0.05

*CD, Crohn's disease; HC, healthy control; CDF, Crohn's disease female; HCF, healthy control female; t, t-value; p, p-value; r CD, mean bivariate correlation for CD group; r HC, mean bivariate correlation for HC group; R, right; L, left; FP, frontoparietal network; SN, salience network; DMN, default mode network; LAN, language network, VIS, visual network*.

When comparing CD and HC females (Table 2, Figure 1B), we found increased FC of the SN left anterior insula (AI) with all four FP network nodes, with a reciprocal FC of the right LPFC and PPC nodes with the left AI. In all four cases, negative FC between nodes for the HC was increased to near or above zero for CD. Negative FC between the medial VIS network node and bilateral SN AI were decreased for CD. No differences were found between CD females and CD males, or CD males and HC males. For the CD group, neither Montreal classifications nor disease duration correlated significantly with the FC findings.

### ICA Results

**Figure 2:**
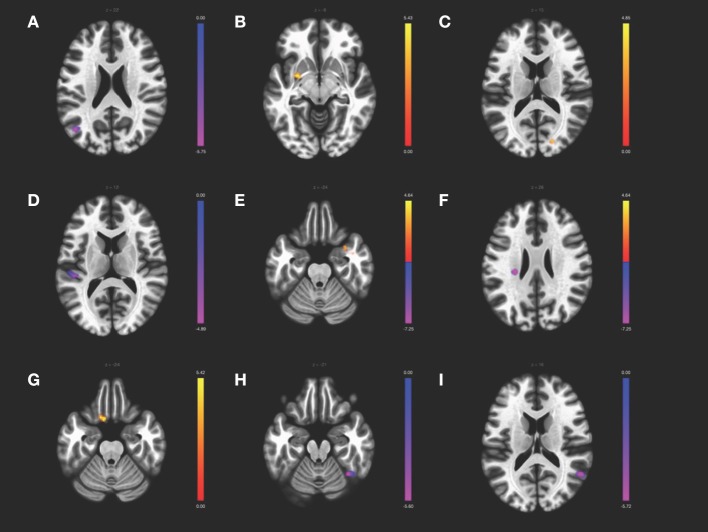
ICA contrast between CD > HC for **(A)** the cerebellar network FC with left superior lateral occipital, **(B)** the visual network FC with left putamen and **(C)** the right occipital pole, and **(D)** the salience network FC with the left planum temporale. The female CD > HC contrast revealed altered FC in the **(E)** DMN with the right temporal pole and **(F)** left insula, **(G)** the SMN with left orbitofrontal cortex, and **(H)** the visual network with the fusiform gyrus, whereas the male CD > HC contrast showed altered FC of **(I)** the DMN with the right angular gyrus. All slices correspond to the peak activation coordinates with the color bar representing positive *t*-values in orange and negative *t*-values in blue. Slices are shown in neurological orientation (left hemisphere on the left of each slice, anterior toward the top of each slice) and are displayed at *T* threshold = 3.5, uncorrected height threshold *p* < 0.001, and FDR-corrected cluster threshold *p* < 0.05.

Results from the ICA analysis (Table 3, Figure 2) show FC decrease from positive to negative between the CER network with superior lateral occipital cortex and SN with left planum temporale in CD patients compared to HCs. CD patients also showed an increase in FC from negative to positive between the VIS network and the left putamen and right occipital pole compared to HCs. Sex differences were observed for the ICA. CD females, as compared with HC females, showed multiple differences, including a decrease from positive to negative FC of the DMN with the left insula and of the VIS with the right fusiform gyrus, and an increase in FC from negative to positive correlation of the DMN with the right temporal pole and of the SMN with the left orbitofrontal cortex. CD males showed a decrease in positive FC of the DMN with the right angular gyrus, as compared to the HC males. There was no significant difference between the sexes within the CD group. No significant correlations were found for Montreal classifications or disease duration.

**Table 3:** Differences in FC between Crohn's disease patients and healthy controls (CD > HC) controlling for age, sex, BMI, and acquisition site, and between CD and HC females (CDF > HCF), and CD and HC males (CDM > HCM) controlling for age, BMI, and acquisition site (*T* threshold = 3.5, uncorrected height threshold *p* < 0.001, FDR-corrected cluster threshold *p* < 0.05).

**Network**	**Region**	**Voxels**	**Peak MNI coordinates**			***t***	***p***	**CD**	**HC**
			***x***	***y***	***z***				
**CD > HC**									
CER	Superior lateral occipital L	137	−46	−68	22	−5.75	0.005	−0.53	0.81
VIS	Putamen L	97	−30	00	−8	5.43	0.029	0.77	−0.70
	Occipital pole R	126	18	−82	14	4.91	0.007	1.40	−0.57
SN	Planum temporale L	113	−46	−30	12	−4.96	0.020	−0.34	1.36
**CDF > HCF**									
DMN	Temporal pole R	89	32	8	−24	4.64	0.038	0.55	−2.76
	Insula L	103	−28	−24	26	−7.25	0.038	−0.80	2.70
SMN	Orbital frontal cortex L	135	−8	14	−26	5.42	0.012	0.92	−2.94
VIS	Fusiform cortex R	124	36	−54	−22	−5.38	0.011	−0.48	1.03
**CDM > HCM**									
DMN	Angular gyrus R	106	56	−56	16	−5.72	0.025	1.18	3.40

*The number of voxels per cluster, MNI coordinates of the peak intensity voxel per cluster, t, p, and mean values are given*.

*CD, Crohn's disease; HC, healthy control; CDF, Crohn's disease female; HCF, healthy control female; CDM, Crohn's disease male; HCM, healthy control male; R, right; L, left; CER, cerebellar network; VIS, visual network; SN, salience network; DMN, default mode network; SMN, sensorimotor network*.

### *Post-hoc* Multimodal Analysis

Areas of spatial overlap were identified for the combinations of the ICA and ROI-to-ROI, the ICA and VBM, and the ROI-to-ROI and VBM layers, as well as for the combination of all three layers. The overlay of the two functional results showed spatial overlap in bilateral supplementary motor area, right parahippocampal gyrus, and left insula and thalamus. The overlay of the ICA and VBM layers showed spatial overlap in bilateral supplementary motor area, right cerebellum, and left insula, thalamus, and hippocampus. Overlay of the ROI-to-ROI and VBM layers showed spatial overlap in bilateral cerebellum and supplementary motor area, and left paracentral lobule, fusiform gyrus, insula, and rolandic operculum. Spatial overlap of all three layers was observed for bilateral supplementary motor area and a region spanning the left insula and rolandic operculum.

## Discussion

This study compared the structural and functional brain images of patients with CD and HC. An ROI-to-ROI analysis of network nodes displayed increased FC between nodes of cognitive control (frontoparietal network) and salience networks, and decreased FC within nodes of the DMN for the CD group as compared to the HC. Using ICA, we identified altered network FC in the cerebellar, visual, and salience networks. A preliminary look at the effect of CD that differs based on sex suggests that the alterations in the DMN are driven by the males in the study while the frontoparietal, salience, and visual network alterations appear driven by the females. A multimodal assessment of the spatial overlap of the structural and two functional results indicated that bilateral supplementary motor area and left insula were common to all three analyses.

The ROI analysis revealed that pre-frontal cortex FC is altered in CD. The FC of the frontoparietal node, right LPFC, is increased reciprocally with bilateral supramarginal gyrus salience network nodes. The frontoparietal network is a cognitive control network involved in modulating attention and expectancy-induced pain modulation. Within the DMN, MPFC is decreased reciprocally with the left LP cortex. Altered pre-frontal cortex FC is consistent with previous studies of CD including differences in amplitude of low-frequency fluctuations (29), in regional homogeneity for CD patients without pain (28), and in relation to effective treatment (30). The altered FC between anterior (MPFC) and posterior (LP) nodes within the DMN is also consistent with previous work (29). Although reduced GM volume of MPFC for CD as compared to HC has also been reported (23), this finding was not reproduced in the current study. The involvement of pre-frontal cortex changes in CD may be explained by its role in mediating antinociceptive effects and modulation of pain (37). MPFC–nucleus accumbens connectivity has been linked to the chronification of pain (37). An investigation into pre-frontal cortex alterations and its associations with pain, psychiatric comorbidity, and cognitive deficits within CD is warranted. As participants in the current study were screened for depression or anxiety as exclusion criteria and no cognitive measures were obtained, the associations between brain alterations and psychiatric comorbidity or cognition cannot be assessed. The exclusion of CD patients with psychiatric comorbidity facilitated the interpretation of the effects of CD on the brain. Future work contrasting CD patients with and without comorbidities will prove informative regarding brain changes associated with the disease.

The increased FC between the frontoparietal and salience network nodes is suggestive of stronger ties between cognitive control and meaningful external/internal stimulus detection. It has been proposed that the salience network acts as a “switch” between task-positive networks, such as the frontoparietal network involved in cognitive control (38) and the task-negative network involved in internal mentation, the DMN (39). It is possible that the stronger coupling of awareness and cognitive process is a reflection of the physical discomfort and the adverse effect that CD has on cognition (40). The decreased within-network FC of the DMN may indicate that the CD patients experience disrupted mind-wandering or self-referential thought. The enhanced functional coupling of the salience network “switch” and frontoparietal network cognitive control, combined with the reduced DMN integrity, underlies this interpretation.

Whereas the ROI-to-ROI analysis calculates only the FC between each of the 32 network nodes, the ICA performs at a voxel-to-voxel level assessing the FC of the entire brain. ICA has the advantage of identifying functional connections of brain regions not restricted to the boundaries of the network nodes, within each of the components associated with RSNs. The ICA results revealed differences between CD and HC in three RSNs that, taken together, suggest alterations in sensorimotor processes. The cerebellar network showed decreased FC with the superior lateral occipital cortex, a brain region involved in visual processing. Increased FC was observed for the visual network and the occipital pole, a region involved in vision. FC of the salience network with the left planum temporale, a region involved in locating sound in space, was decreased. This may indicate an alteration of the salience network, which detects salient stimuli from the environment, with an auditory network; however, this cannot be known from the current study as no auditory network was investigated. Increased FC was observed within the visual network component with the putamen, a region involved in movement, decision-making, and learning, which may correspond to an alteration of sensorimotor and behavioral processes related to CD symptoms. It is possible that for persons with CD, sensitivity to visceral sensory information, and modulation of a physical response to that information, is more acute and the need to learn and adapt behavior accordingly may be greater than for HC. As examples, the necessity of strategically planned outings or an urgent need to locate a bathroom while out may be stressors for persons living with a disease that include abdominal pain and diarrhea as common symptoms. The increased co-activity of brain regions involved in sensorimotor and executive functions may make sense in this context.

In an analysis of whether the group level results were related to sex, it became apparent that the females in the study largely drove the frontoparietal, salience and visual network differences whereas the males drove the DMN alteration. Although no differences were detected in the ROI analysis between CD females and males, or CD males and HC males, numerous differences between CD and HC females were observed. Notably, the CD females had increased FC between frontoparietal and salience network nodes. FC was increased in the right frontoparietal nodes, LPFC and PPC, with the salience node left anterior insula that, in return, had increased FC with all four frontoparietal nodes, bilateral LPFC and PPC. The CD females had decreased FC of both the medial and occipital visual network nodes with numerous other salience network nodes. As no differences in FC were observed for the frontoparietal, salience, or visual network nodes for the males, a trend emerges that suggests the females in this study may possibly drive these network changes.

Curiously, there were no group differences in the ICA for the frontoparietal or DMN components, as there were for the ROI analysis. However, when investigating sex differences for each of the RSNs, there did appear to be an explanation for this: CD males, as compared to HC males, showed a decrease in FC for the DMN with the angular gyrus, whereas CD females as compared to HC females showed decreased FC for the left insula and increased FC for the right temporal pole for the DMN component. Whereas, the males showed decreased FC *within* the DMN, the females showed altered FC of the DMN with brain regions *outside* of the network. Given that the DMN was functionally connected to disparate brain regions for each sex may account for the lack of significant difference in the ICA at the group level. It is worth noting that the decreased FC of the DMN with the angular gyrus is consistent with the ROI analysis, which showed a decrease in FC between the DMN nodes MFPC and LP—in fact, the angular gyrus and LP cortex share some of the same coordinates but are labeled differently depending on the atlas used. Therefore, the ICA results support the ROI results and, further, suggest that the males in the study are behind this observed alteration. The multimodal analysis showed spatial overlap of the ICA and ROI layers in a medial frontal region, the bilateral supplementary motor areas, although not in MPFC. The left insula was also shown to have spatial overlap for the ICA and ROI layers (in fact, both supplementary motor area and insula have spatial overlap for the overlay of the ICA, ROI, and VBM layers, although the VBM layer represents an uncorrected t-map and therefore should be interpreted with caution). According to the sex differences observed in the ICA and ROI analyses, it would appear that the males might contribute to the medial frontal region overlap whereas the females contribute to the insula overlap, in the multimodal analysis of group-level results. However, the subgroups of males and females are small and a larger dataset is required to test this idea.

ICA results show CD females had decreased FC of the visual network with the right temporal occipital fusiform gyrus, which supports the multiple ROI findings of altered visual network FC for CD females. Although perhaps not intuitive that visual network changes would be involved in CD, differences in visual processing regions have been reported in previous CD studies, including cortical thickness differences in left lateral occipital cortex (24), hypergyrification of left lingual gyrus (41) for CD, and increased FC within visual medial and frontoparietal networks in IBD (42), and similar reports are seen for other chronic conditions, such as knee osteoarthritis (43), persistent somatoform pain disorder (44), postherpetic neuralgia (45), migraine (46), and fibromyalgia (47). Having recognized the plethora of studies reporting visual system FC alterations in chronic pain populations, Shen et al. (48) investigated the FC of visual network nodes in a chronic low back pain population using an ROI analysis and a support vector machine classifier. Significant FC alterations were found for primary and bilateral dorsal visual network seeds with somatosensory and motor brain regions and a classification accuracy of 79.3% was reported for distinguishing chronic low back pain from HC. The authors proposed an adaptation or self-adjustment mechanism and cross-modal interaction between visual, somatosensory, motor, attention, and saliency networks to account for their findings (48). The results presented in the current study are supportive of cross-modal interactions between these networks.

It is important to note the study limitations. This is a preliminary study with a modest sample size necessitating replication in future studies. Greater sample size may be required for detecting GM volume differences in particular. Regarding the ROI results, the FC decrease in the DMN with the insula is consistent with reports of increased anticorrelations between salience network AI and DMN in osteoarthritis (49) and decreased FC between MPFC and AI in IBS (50); however, the finding in the current study must be interpreted with caution as the peak coordinate was in insula but the majority of the cluster voxels were in non-labeled atlas coordinates. Similarly, the results of the multimodal analyses revealed the insula to have spatial overlap among layers, but this should be interpreted with caution given that the VBM layer displayed results uncorrected for multiple comparisons.

In summary, this study reports on a ROI analysis that shows increased FC between the cognitive control and salience networks and decreased within the DMN for persons with CD. Both results have altered pre-frontal cortex FC for CD. The ICA results show differences in cerebellar connectivity and visual and auditory processing regions that require further investigation. Contrasts based on sex revealed that CD males and females might differ in how their disease affects their RSN FC. This preliminary analysis will be instrumental in guiding hypotheses for future work.

## Data Availability Statement

The datasets for this article are not publicly available due to restrictions of institutional research ethics regarding confidential/identifiable human data. Requests to access the datasets should be directed to Jennifer Kornelsen: jennifer.kornelsen@umanitoba.ca.

## Ethics Statement

The studies involving human participants were reviewed and approved by University of Manitoba Research Ethics Board and UCLA Institutional Review Board. The patients/participants provided their written informed consent to participate in this study.

## Author Contributions

JK, JL, EM, and CB contributed to the conception and design of the study. KW organized the database. AW and JK performed the statistical analysis. AW wrote the first draft of the manuscript. JK wrote the sections of the manuscript. All authors contributed to the manuscript revision, read, and approved the submitted version.

### Conflict of Interest

The authors declare that the research was conducted in the absence of any commercial or financial relationships that could be construed as a potential conflict of interest.

## References

[1] Torres J Mehandru S Colombel J-F Peyrin-Biroulet L. Crohn's disease. Lancet. (2017) 389:1741–55. 10.1016/S0140-6736(16)31711-1 27914655

[2] Ng SC Shi HY Hamidi N Underwood FE Tang W Benchimol EI. Worldwide incidence and prevalence of inflammatory bowel disease in the 21st century: a systematic review of population-based studies. Lancet. (2017) 390:2769–78. 10.1016/S0140-6736(17)32448-0 29050646

[3] Lerebours E Gower-Rousseau C Merle V Brazier F Debeugny S Marti R. Stressful life events as a risk factor for inflammatory bowel disease onset: a population-based case–control study. Am J Gastroenterol. (2007) 102:122–31. 10.1111/j.1572-0241.2006.00931.x 17100973

[4] Long MD Drossman DA. Inflammatory bowel disease, irritable bowel syndrome, or what: a challenge to the functional-organic dichotomy. Am J Gastroenterol. (2010) 105:1796–8. 10.1038/ajg.2010.162 20686466

[5] Cámara RJA Schoepfer AM Pittet V Begré S von Känel R. Mood and nonmood components of perceived stress and exacerbation of crohn's disease. Inflamm Bowel Dis. (2011) 17:2358–65. 10.1002/ibd.21623 21287671

[6] Farrokhyar F Marshall JK Easterbrook B Irvine EJ. Functional gastrointestinal disorders and mood disorders in patients with inactive inflammatory bowel disease: prevalence and impact on health. Inflamm Bowel Dis. (2006) 12:38–46. 10.1097/01.MIB.0000195391.49762.89 16374257

[7] Rampton D. Does stress influence inflammatory bowel disease? Dig Dis. (2009) 27(Suppl. 1):76–9. 10.1159/000268124 20203500

[8] Marrie RA Walld R Bolton JM Sareen J Walker JR Patten SB. Increased incidence of psychiatric disorders in immune-mediated inflammatory disease. J Psychosom Res. (2017) 101:17–23. 10.1016/j.jpsychores.2017.07.015 28867419

[9] Walker JR Ediger JP Graff LA Greenfeld JM Clara I Lix L. The manitoba IBD cohort study: a population-based study of the prevalence of lifetime and 12-month anxiety and mood disorders. Am J Gastroenterol. (2008) 103:1989–97. 10.1111/j.1572-0241.2008.01980.x 18796096

[10] Bonaz BL Bernstein CN. Brain-gut interactions in inflammatory bowel disease. Gastroenterology. (2013) 144:36–49. 10.1053/j.gastro.2012.10.003 23063970

[11] Mayer Emeran A Tillisch K. The brain-gut axis in abdominal pain syndromes. Annu Rev Med. (2011) 62:381–96. 10.1146/annurev-med-012309-103958 21090962 PMC3817711

[12] Soares JM Sampaio A Ferreira LM Santos NC Marques P Marques F. Stress impact on resting state brain networks. PLoS ONE. (2013) 8:e66500. 10.1371/journal.pone.0066500 23840493 PMC3686683

[13] Kim P Evans GW Angstadt M Ho SS Sripada CS Swain JE. Effects of childhood poverty and chronic stress on emotion regulatory brain function in adulthood. Proc Natl Acad Sci USA. (2013) 110:18442–7. 10.1073/pnas.1308240110 24145409 PMC3831978

[14] Thomason ME Hamilton JP Gotlib IH. Stress-induced activation of the HPA axis predicts connectivity between subgenual cingulate and salience network during rest in adolescents. J Child Psychol Psychiatr. (2011) 52:1026–34. 10.1111/j.1469-7610.2011.02422.x 21644985 PMC3169772

[15] Kim ES Cho KB Park KS Jang BI Kim KO Jeon SW. Predictive factors of impaired quality of life in Korean and mood disorders. J Clin Gastroenterol. (2013) 47:38–44. 10.1097/MCG.0b013e318266fff5 23090047

[16] Tagliazucchi E Balenzuela P Fraiman D Chialvo DR. Brain resting state is disrupted in chronic back pain patients. Neurosci Lett. (2010) 485:26–31. 10.1016/j.neulet.2010.08.053 20800649 PMC2954131

[17] Tian T Guo L Xu J Zhang S Shi J Liu C. Brain white matter plasticity and functional reorganization underlying the central pathogenesis of trigeminal neuralgia. Sci Rep. (2016) 6:36030. 10.1038/srep36030 27779254 PMC5078771

[18] Yoshino A Okamoto Y Okada G Takamura M Ichikawa N Shibasaki C. Changes in resting-state brain networks after cognitive–behavioral therapy for chronic pain. Psychol Med. (2018) 48:1148–56. 10.1017/S0033291717002598 28893330

[19] Femenía T Gómez-Galán M Lindskog M Magara S. Dysfunctional hippocampal activity affects emotion and cognition in mood disorders. Brain Res. (2012) 1476:58–70. 10.1016/j.brainres.2012.03.053 22541166

[20] Wiebking C Bauer A De Greck M Duncan NW Tempelmann C Northoff G. Abnormal body perception and neural activity in the insula in depression: an fMRI study of the depressed material me. World J Biol Psychiatr. (2010) 11:538–49. 10.3109/15622970903563794 20146653

[21] Bär K-J Wagnera G Koschke M Boettger S Boettger MK Schlösser R. Increased prefrontal activation during pain perception in major depression. Biol Psychiatr. (2007) 62:1281–7. 10.1016/j.biopsych.2007.02.011 17570347

[22] Agostini A Benuzzi F Filippini N Bertani A Scarcelli A Farinelli V. New insights into the brain involvement in patients with crohn's disease: a voxel-based morphometry study. Neurogastroenterol Motil. (2013) 25:147–82. 10.1111/nmo.12017 22998431

[23] Bao CH Liu P Liu HR Wu LY Shi Y Chen WF. Alterations in brain grey matter structures in patients with crohn's disease and their correlation with psychological distress. J Crohn's Colitis. (2015) 9:532–40. 10.1093/ecco-jcc/jjv057 25895879

[24] Nair VA Beniwal-Patel P Mbah I Young BM Prabhakaran V Saha S. Structural imaging changes and behavioral correlates in patients with crohn's disease in remission. Front Hum Neurosci. (2016) 10:460. 10.3389/fnhum.2016.00460 27695405 PMC5025433

[25] Thomann AK Griebe M Thomann PA Hirjak D Ebert MP Szabo K. Intrinsic neural network dysfunction in quiescent crohn's disease. Sci Rep. (2017) 7:978–80. 10.1038/s41598-017-11792-y 28912568 PMC5599642

[26] Thomann AK Griebe M Thomann PA Reindl W Wolf C Altered intrinsic brain function in crohn's disease. J Crohn's Colitis. (2017) 11(Suppl. 1):S126–7. 10.1093/ecco-jcc/jjx002.225

[27] Agostini A Ballotta D Righi S Moretti M Bertani A Scarcelli A. Stress and brain functional changes in patients with crohn's disease: a functional magnetic resonance imaging study. Neurogastroenterol Motil. (2017) 29:1–10. 10.1111/nmo.13108 28560758

[28] Bao CH Liu P Liu HR Wu LY Jin XM Wang SY. Differences in regional homogeneity between patients with crohn's disease with and without abdominal pain revealed by resting-state functional magnetic resonance imaging. Pain. (2016) 157:1037–44. 10.1097/j.pain.0000000000000479 26761381 PMC4969077

[29] Bao C Liu P Liu H Jin X Shi Y Wu L. Difference in regional neural fluctuations and functional connectivity in crohn's disease: a resting-state functional MRI study. Brain Imaging Behav. (2018) 12:1795–803. 10.1007/s11682-018-9850-z 29464530 PMC6218319

[30] Bao C Liu P Liu H Jin X Calhoun VD Wu L. Different brain responses to electro-acupuncture and moxibustion treatment in patients with crohn's disease. Sci Rep. (2016) 6:36636. 10.1038/srep36636 27857211 PMC5114555

[31] Liu P Li R Bao C Wei Y Fan Y Liu Y. Altered topological patterns of brain functional networks in crohn's disease. Brain Imaging Behav. (2018) 12:1466–78. 10.1007/s11682-017-9814-8 29297154

[32] Hou J Mohanty R Nair VA Dodd K Beniwal-Patel P Saha S. Alterations in resting-state functional connectivity in patients with crohn's disease in remission. Sci Rep. (2019) 9:7412. 10.1038/s41598-019-43878-0 31092855 PMC6520362

[33] Evertsz' FB Hoeks CCMQ Nieuwkerk PT Stokkers PCF Ponsioen CY Bockting CLH Development of the patient harvey bradshaw index and a comparison with a clinician-based harvey bradshaw index assessment of crohn's disease activity. J Clin Gastroenterol. (2013) 47:850–6. 10.1097/MCG.0b013e31828b2196 23632348

[34] Satsangi J Silverberg MS Vermeire S Colombel JF. The montreal classification of inflammatory bowel disease: controversies, consensus, and implications. Gut. (2006) 55:749–53. 10.1136/gut.2005.082909 16698746 PMC1856208

[35] Ashburner J. A fast diffeomorphic image registration algorithm. Neuroimage. (2007) 38:95–113. 10.1016/j.neuroimage.2007.07.007 17761438

[36] Whitfield-Gabrieli S Nieto-Castanon A. Conn: a functional connectivity toolbox for correlated and anticorrelated brain networks. Brain Connect. (2012) 2:125–41. 10.1089/brain.2012.0073 22642651

[37] Ong WY Stohler CS Herr DR. Role of the prefrontal cortex in pain processing. Mol Neurobiol. (2019) 56:1137–66. 10.1007/s12035-018-1130-9 29876878 PMC6400876

[38] Marek S Dosenbach NUF. The frontoparietal network: function, electrophysiology, and importance of individual precision mapping. Dial Clin Neurosci. (2018) 20:133–40. 30250390 10.31887/DCNS.2018.20.2/smarek PMC6136121

[39] Sridharan D Levitin DJ Menon V. A critical role for the right fronto-insular cortex in switching between central-executive and default-mode networks. Proc Natl Acad Sci USA. (2008) 105:12569–74. 10.1073/pnas.0800005105 18723676 PMC2527952

[40] Whitehouse CE Fisk JD Bernstein CN Berrigan LI Bolton JM Graff LA Comorbid anxiety, depression, and cognition in MS and other immune-mediated disorders. Neurology. (2019) 92:E406–17. 10.1212/WNL.0000000000006854 PMC6369907 30635487

[41] Thomann AK Thomann PA Wolf RC Hirjak D Schmahl C Ebert MP Altered markers of brain development in crohn's disease with extraintestinal manifestations—a pilot study. PLoS ONE. (2016) 11:e0163202 10.1371/journal.pone.0163202 27655165 PMC5031401

[42] Neeb L Bayer A Bayer KE Farmer A Fiebach JB Siegmund B. Transcranial direct current stimulation in inflammatory bowel disease patients modifies resting-state functional connectivity: a RCT. Brain Stimul. (2019) 12:978–80. 10.1016/j.brs.2019.03.001 30905546

[43] Pujol J Martínez-Vilavella G Llorente-Onaindia J Harrison BJ López-Solà M López-Ruiz M. Brain imaging of pain sensitization in patients with knee osteoarthritis. Pain. (2017) 158:1831–8. 10.1097/j.pain.0000000000000985 28683024

[44] Zhao Z Huang T Tang C Ni K Pan X Yan C. Altered resting-state intra- and inter- network functional connectivity in patients with persistent somatoform pain disorder. PLoS ONE. (2017) 12:e0176494. 10.1371/journal.pone.0176494 28453543 PMC5409184

[45] Cao S Qin B Zhang Y Yuan J Fu B Xie P. Herpes zoster chronification to postherpetic neuralgia induces brain activity and grey matter volume change. Am J Transl Res. (2018) 10:184–99. 29423004 PMC5801357

[46] Liu J Zhao L Li G Xiong S Nan J Li J. Hierarchical alteration of brain structural and functional networks in female migraine sufferers. PLoS ONE. (2012) 7:e51250. 10.1371/journal.pone.0051250 23227257 PMC3515541

[47] Pujol J Macià D Garcia-Fontanals A Blanco-Hinojo L López-Solà M Garcia-Blanco S. The contribution of sensory system functional connectivity reduction to clinical pain in fibromyalgia. Pain. (2014) 155:1492–503. 10.1016/j.pain.2014.04.028 24792477

[48] Shen W Tu Y Gollub RL Ortiz A Napadow V Yu S. Visual network alterations in brain functional connectivity in chronic low back pain: a resting state functional connectivity and machine learning study. NeuroImage Clin. (2019) 22:101775. 10.1016/j.nicl.2019.101775 30927604 PMC6444301

[49] Cottam WJ Iwabuchi SJ Drabek MM Reckziegel D Auer DP. Altered connectivity of the right anterior insula drives the pain connectome changes in chronic knee osteoarthritis. Pain. (2018) 159:929–38. 10.1097/j.pain.0000000000001209 29557928 PMC5916486

[50] Qi R Liu C Ke J Xu Q Zhong J Wang F. Intrinsic brain abnormalities in irritable bowel syndrome and effect of anxiety and depression. Brain Imaging Behav. (2016) 10:1127–34. 10.1007/s11682-015-9478-1 26556814

